# Electronically Tunable Differential Integrator: Linear Voltage Controlled Quadrature Oscillator

**DOI:** 10.1155/2015/690923

**Published:** 2015-04-19

**Authors:** Rabindranath Nandi, Sandhya Pattanayak, Palaniandavar Venkateswaran, Sagarika Das

**Affiliations:** ^1^Department of Electronics & Telecommunication Engineering, Jadavpur University, Kolkata 700032, India; ^2^Department of Electronics & Communication Engineering, Narula Institute of Technology, Kolkata 700109, India; ^3^Department of Electronics Engineering, B. P. P. Institute of Technology, Kolkata 700052, India

## Abstract

A new electronically tunable differential integrator (ETDI) and its extension to voltage controlled quadrature oscillator (VCQO) design with linear tuning law are proposed; the active building block is a composite current feedback amplifier with recent multiplication mode current conveyor (MMCC) element. Recently utilization of two different kinds of active devices to form a composite building block is being considered since it yields a superior functional element suitable for improved quality circuit design. The integrator time constant (*τ*) and the oscillation frequency (*ω*_*o*_) are tunable by the control voltage (*V*) of the MMCC block. Analysis indicates negligible phase error (*θ*_*e*_) for the integrator and low active *ω*_*o*_-sensitivity relative to the device parasitic capacitances. Satisfactory experimental verifications on electronic tunability of some wave shaping applications by the integrator and a double-integrator feedback loop (DIFL) based sinusoid oscillator with linear *f*_*o*_ variation range of 60 KHz~1.8 MHz at low THD of 2.1% are verified by both simulation and hardware tests.

## 1. Introduction

Dual-input integrators with electronic tunability are useful functional components for numerous analog signal processing and waveforming applications [1]. A number of single and dual-input passive tuned integrators using various active building blocks are available [2, 3].

However, integrators with electronically adjustable time constant (*τ*) find some exclusive applications [4–6], for example, in electronically tunable biquadratic phase selective filter design [7], as electronic reset controller and phase compensator [8] for process control loops.

Quadrature sinusoid oscillators are widely used in orthogonal signal mixers, in PLLs and SSB modulators; some such oscillators based on various active devices, for example, voltage operational amplifier-VOA [9], CFOA [10, 11], CDBA [12, 13], CDTA [14], DDCC [15], CCCCTA [16], OTA [17], and DVCCTA [18, 19], are reported.

Here we present a simple electronically tunable dual-input integrator (ETDI) topology based on a composite current feedback amplifier- (CFA-) multiplication mode current conveyor (MMCC) building block. It has been pointed out in the recent literature [20, 21] that utilization of two different kinds of active elements to form a composite building block yields superior functional result in analog signal processing applications.

Hence the topic of quadrature sinusoidal oscillator design and implementation with better quality is receiving considerable research interest at present. A number of such oscillators using various active building blocks [9–17] are now available. Here, we present a new simple ETDI topology, based on a composite CFA-MMCC building block with grounded capacitor; subsequently, a double-integrator feedback loop (DIFL), with one inverting and the other noninverting, is utilized to design a linear VCQO wherein a pair of grounded *RC*-section selects the appropriate signal generation band and the control voltage (*V*) of the MMCC tunes oscillation frequency (*f*_*o*_) linearly; no component matching constraint is involved here. This current conveyor element is quite an elegant building block with a dedicated control voltage (*V*) terminal; hence the MMCC is a versatile active component suitable for electronically tunable function circuit design.

It is seen in recent literature that such linear VCQO is a useful functional block which finds wide range of applications in emerging fields; namely, in certain telemetry-related areas it could convert a transducer voltage to a proportional frequency which is then modulated for subsequent processing [22], as quantizer for frequency-to-digital or time-to-digital conversion [23] and also as the spectrum monitor receiver [24] in cognitive radio communication studies.

Here, we present the design and realization of a new linear VCQO using the composite type active device; analysis shows that device imperfections, namely, port tracking errors (|*ε*| ≪ 1) and parasitic capacitors (*C*_*z*,*m*_) at current source nodes, yield negligible effects on the nominal design, whereby the active-sensitivity figures are extremely low. Experimental measurements by simulation and hardware tests on the proposed design indicate satisfactory results with *f*_*o*_-tunability in the range 60 KHz–1.8 MHz following the variation of a suitable control voltage (1 ≤ *V*(d.c.volt) ≤ 10) wherein a desired band-spread may be selected by appropriate choice of the grounded *RC* components [25], without any component matching constraint, even with nonideal devices.

## 2. Analysis

**Figure 1:**
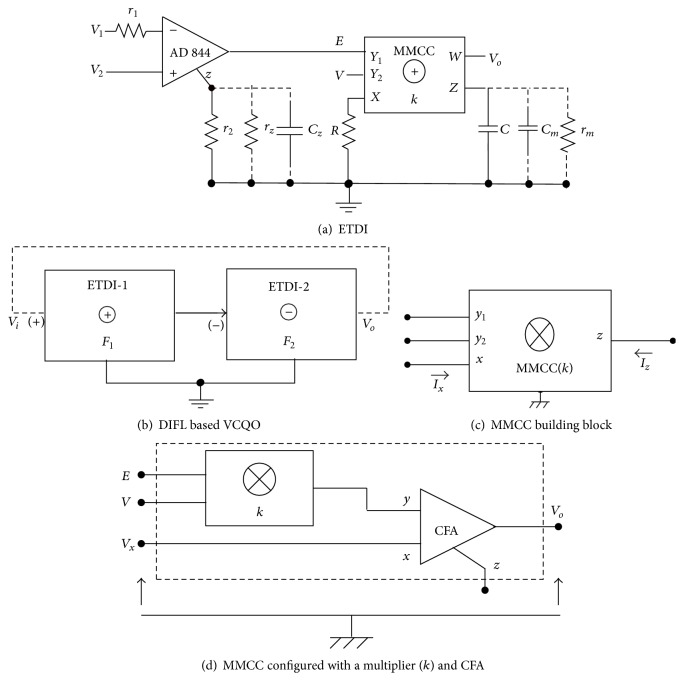
Proposed electronically tunable functional circuits.

The ETDI topology is shown in Figure 1(a); the nodal relations of the active blocks are *i*_*z*_ = *α*_1_ *i*_*x*_, *v*_*x*_ = *β*_1_ *v*_*y*_, *E* = *δ*_1_ *v*_*z*_, and  *i*_*y*_ = 0 for the CFA and *i*_*z*_ = *α*_2_ *i*_*x*_, *v*_*x*_ = *β*_2_ *kEV*, *v*_*o*_ = *δ*_2_ *v*_*z*_, and *i*_*y*1_ = 0 = *i*_*y*2_ for the MMCC where *k*( = 0.1/volt) is multiplication constant [12] and *V* is control voltage. The port transfer ratios (*α*, *β*, and *δ*) are unity for ideal elements; the imperfections may be postulated in terms of some small error coefficients (.01 ≤ |*ε*| ≤ .04) as *α* ≈ (1 − *ε*_*i*_), *β* ≈ (1 − *ε*_*v*_), and *δ* ≈ (1 − *ε*_*o*_). Also, shunt-*rC* parasitic components appear [26–28] at the *z*-node of the blocks having typical values in the range of 3 pF ≤ *C*_*z*,*m*_ ≤ 6 pF and 2 M*Ω* ≤ *r*_*z*,*m*_ ≤ 5 M*Ω*; since resistance values used in the design are in KΩ ranges, their ratios to *r*_*z*,*m*_ are extremely small and hence effects of *r*_*z*,*m*_ are negligible in the design. It may also be mentioned that a low-value internal parasitic resistance (*r*_*x*_ ≈ 45 *Ω*) appears in series with the current path at *x*-node of the devices; its effect can be minimized by absorbing *r*_*x*_-value in the load resistors at these nodes. Routine analysis assuming *V*_in_ = (*β*_1_ *V*_2_ − *V*_1_) yields the open-loop transfer *F* ≡ *V*_*o*_/*V*_in_ in Figure 1(a) as

(1) $$\begin{array}{c} F=\frac{\mu n}{\left\{s\tau_{o}\left(1+m\right)+\eta \right\}\left\{\left(s\tau_{z}+1+\sigma \right)\right\}}, \end{array}$$

where

(2) μ=α1α2β2δ1δ2≈1−εt,εt=εi1+εi2+εv2+εo1+εo2,n=r2r1,  m=CmC≪1,  τz=Czr2,η=Rrm≪1,  σ=r2rz≪1,ωωz≪1; ωz=1τz,τo=10RCV;  k=0.1volt−1.

It may be seen that effect of *C*_*m*_ may be compensated by absorbing its value in *C* since both are grounded [25], an attractive feature for microminiaturization. The noninverting input signal is also slightly altered; in practice, however, the deviation is quite negligible as we observed during the experimental verification.

The port errors alter the magnitude response slightly by a factor (1 − *ε*_*t*_); however, a phase error (*θ*_*e*_) is introduced at relatively higher side of frequency *ω*_*z*_( = 1/*τ*_*z*_), given by *θ*_*e*_ = arctan(*ω*/*ω*_*z*_). Since *C*_*z*_ ≪ *C*, *ω*/*ω*_*z*_ ≪ 1; that is, *θ*_*e*_ ≈ 0; typical values [27] indicate that if *C*_*z*_ ≈ 4 pF, then *f*_*z*_ ≈ 41 MHz with *r*_2_ ≈ 1 KΩ (typical) which yields *θ*_*e*_ ≈ 3° at 2.5 MHz around the nominal phase of *π*/2. The quality factor (*q*) of the integrator is derived as *q* ≈ (1/tan*θ*_*e*_) ≫ 1.

Hence, it is seen that the effects of device nonidealities are quite negligible; assuming therefore that *ε* ≈ 0 and *r*_2_ = *r*_1_ for simplicity, we get the desired ETDI transfer (*F*) from (1) as

(3) $$\begin{array}{c} F\equiv \frac{V_{o}}{\left(V_{2}-V_{1}\right)}=\frac{1}{s\tau_{o}}. \end{array}$$

## 3. Linear VCQO Design

The proposed oscillator is designed with DIFL using the block diagram of Figure 1(b); neglecting port errors (*ε* ≈ 0) in (1), we get the loop-gain (*F*_*o*_ ≡ *F*_1_ *F*_2_) of the DIFL, assuming *m* ≪ 1 and *n* = 1, as

(4) $$\begin{array}{c} F_{o}=-{\left\{s^{2}\tau_{o1}\tau_{o2}\left(s\tau_{z1}+1\right)\left(s\tau_{z2}+1\right)\right\}}^{-1}. \end{array}$$

The MMCC block is shown in Figure 1(c).

Total phase shift (Φ) of loop-gain is

(5) $$\begin{array}{c} \Phi =\pi -\left[2\left(\frac{\pi }{2}\right)+\left\{\arctan\left(\frac{\omega }{\omega_{z1}}\right)+\arctan\left(\frac{\omega }{\omega_{z2}}\right)\right\}\right]. \end{array}$$

The parasitic phase components are extremely low since the lossy capacitors (*C*_*z*,*m*_) create distant pole frequencies compared to the usable frequency range; hence the input and output stimulus of the DIFL would be in same phase at unity gain and closure of loop incites sinusoid oscillations build-up; the corresponding characteristic equation is

(6) $$\begin{array}{c} \left(s^{2}\tau_{o1}\tau_{o2}+1\right)=0 \end{array}$$

which yields the oscillation frequency after putting *s* ≡ *jω*, as

(7) $$\begin{array}{c} \omega_{o}\equiv \frac{1}{\sqrt{\left\{\tau_{o1}\tau_{o2}\right\}}}=\frac{kV}{\left\{\sqrt{\left(R_{1}C_{1}R_{2}C_{2}\right)}\right\}}. \end{array}$$

With equal-value *RC* components and *k* = 0.1/volt, (7) yields *f*_*o*_ = *V*/(20*πRC*); thus linear tunability of *f*_*o*_ is obtained by directly applying the control voltage (*V*) of MMCC unit. No additional *g*_*m*_ to current conversion circuitry as compared to previous realizations using OTA [14, 17] is required. The active *ω*_*o*_-sensitivity is calculated as *S*_*ε*_^*ω*_*o*_^ = 0.5*ε*/(1 − *ε*_*t*_) ≪ 0.5. The frequency-stability (Ψ) is derived as Ψ = 1, using the relation Ψ ≡ {ΔΦ/(Δ*u*)}|_*u*=1_ where *u* = *ω*/*ω*_*o*_.

## 4. Experimental Results

The proposed ETDI of Figure 1(a) is built with readily available ADF-844/846 type CFA device [28, 29]; since MMCC [30]-chip is not yet commercially available, we configured it [12, 31] as shown in Figure 1(d), with four-quadrant multiplier (ICL-8013 or AD-534) coupled with a current feedback amplifier (CFA) device (AD-844 or AD-846). The bandwidth of the CFA device is almost independent of the closed loop-gain at high slew rate values [28, 29]. This yields the element to be particularly advantageous for various signal processing/generation applications. Recently, reports on superior versions of the CFA element (OPA-695) have appeared [32] indicating very high slew rate (~2.5 KV/*μ*s) and extended bandwidth (~1.4 GHz).

**Figure 2:**
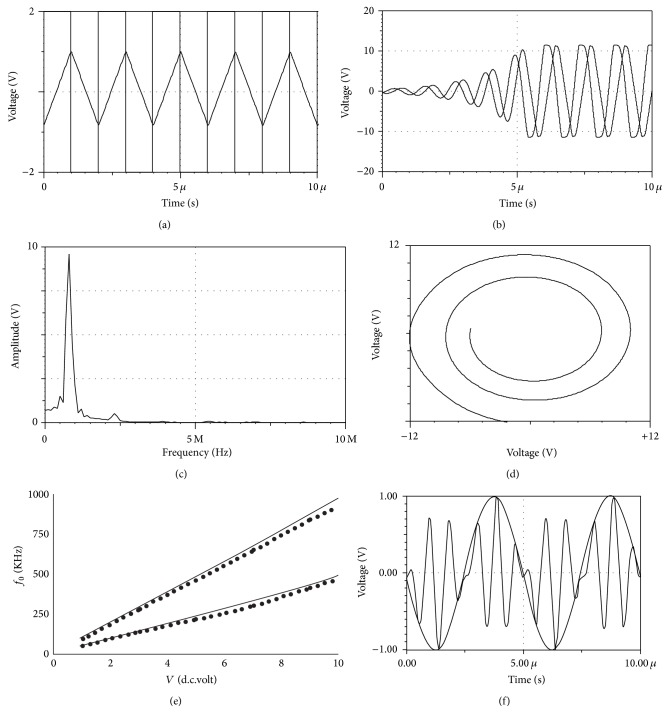
Test responses of proposed ETDI and linear VCQO. (a) Response of integrator with square wave input signals at 500 KHz: *V*_2_ = −*V*_1_ = 2 Volt(pp), *V* = 5 volt, *R* = 1 KΩ, and *C* = 0.5 nF; measured *C*_*z*_ ≈ *C*_*m*_ ≈ 4.2 pF at *V*_cc_ = 0 ± 12 V.d.c. (b) Quadrature oscillator response at the onset of sinusoidal wave build-up at 1.1 MHz with *R* = 1 KΩ, *C* = 165 pF, and *V* = 5 volt. (c) Spectrum of the response in (b). (d) Lissajous pattern of quadrature signals. (e) VCQO tuning characteristics: dotted line: hardware test; firm line: simulation; band-spread selection: upper curve: *R* = 1 KΩ, *C* = 165 pF; lower curve: *R* = 1.5 KΩ, *C* = 220 pF. (f) Quadrature wave modulation: 1.2 MHz input signal *V*_2_ = 2 Vpp to ETDI with *V*_1_ = 0; control voltage = *V* = 2 Vpp at 200 KHz to MMCC.

For the linear VCQO design, we formed the block diagram of Figure 1(b) using one inverting and another noninverting ETDI. The measured responses obtained by simulation and hardware tests are shown in Figure 2; linear *V* to *f*_*o*_ variation characteristics had been verified with 1 ≤ *V*(d.c.volt) ≤ 10 which provided a satisfactory tuning range of up to about 1.8 MHz with appropriate choice of *RC* products.

Practical design responses of proposed circuits have been verified with both ICL-8013 and AD-534 type as multiplier elements, along with both AD-844 and AD-846 type CFA devices; satisfactory results with both sets of components had been verified. A comparative summary of some recent quadrature oscillator characteristics is described in Table 2.

## 5. Some Discussions

Keeping in view the measured responses, a few observations are presented here on functional unit to unit basis; this substantiates the accuracy and versatility of the proposed realization.

*ETDI*

(a) Voltage controlled time constant, either enhancement or tapering adjustment at dual input feature with grounded capacitor; typical time domain response shown in Figure 2(a) verifies the features.
(b) With sinusoid signals, 20 dB/decade frequency roll-off had been observed at 3.3 MHz onwards; measured CMRR ≈ 57 dB at *V*_2_ = −*V*_1_.
(c) Frequency domain phase error is measured as *θ*_*e*_ ≈ 2.4° at 2 MHz; adjustment of *V* for *τ*_*o*_ control did not affect this error. Also the ETDI is practically active-insensitive to port mismatch errors (*ε* ≪ 1) [28, 33].

*CFA-MMCC*

(a) Availability of in-built control voltage node of MMCC adds flexibility to a designer; this feature is verified experimentally by generating a quadrature (integrated) wave modulation response as shown in Figure 2(f). This is a useful application of the CFA-MMCC based ETDI.
(b) Analysis shows that *θ*_*e*_ is dominantly caused by parasitic *C*_*z*_ of CFA device; another component of this error due to MMCC is negligible since *θ*_*e*_(MMCC) ≈ arctan(*ωRC*/*η*) ≈ 0 as *η* ≈ 10^3^.

Thus the overall phase error of *∟F*(*ω*) could be limited to extremely low values for frequencies *ω* ≪ *ω*_*z*_, after selecting moderate values of *r*, while *τ*_*o*_ may be tuned by *V*; these two adjustments are noninteracting.

This substantiates the versatility of selecting a composite building block. Phase error had been measured as in Table 1, with *r* = 1 KΩ and *C*_*z*_ ≈ 4 pF (measured); that is, *f*_*z*_ ≈ 41 MHz.

*DIFL*

(a) Two identical stages, with a common *V* terminal, are cascaded for DIFL topology having nominal phase output of 180°. Tested phase response compounded as *θ*_*e*_(DIFL) ≈ 5.5° at 2 MHz. Loop was observed to be unable to build up oscillation beyond 2 MHz. Therefore parasitic elements tend to limit the usable range of *f*_*o*_ ≤ 2 MHz.

*VCQO*

(a) Literature shows that albeit some quadrature oscillators were presented earlier, very few [10, 17] provide electronically tunable linear *f*_*o*_ tuning law. These designs are based on electronic tuning by a bias current (*I*_*b*_) that is replicated from *g*_*m*_ which requires additional current processing circuitry/hardware consuming extra quiescent power; moreover such *g*_*m*_-to-*I*_*b*_ conversion involves thermal voltage [18] and hence temperature sensitivity issues. In view of these comparative attributes, the proposed design appears to be superior.

## 6. Conclusion

The realization and analysis of a new ETDI and its applicability to the design of linear VCQO using the CFA-MMCC composite building block are presented.

The effects of the device imperfections are examined which are seen to be quite negligible as indicated by low phase and magnitude deviations. The linear *V*-to-*f*_*o*_ tuning characteristics had been experimentally verified in a range of 60 KHz–1.8 MHz with good quality low distortion sine-wave generation response. It may be mentioned that here the linear *f*_*o*_ tuning feature is obtained by the simple and direct application of the same control voltage (*V*) to the appropriate terminal of the MMCC building blocks for the two ETDI stages. Additional current processing circuitry for *g*_*m*_ –to- bias current (*I*_*b*_) conversion and its associated hardware complexity with additional quiescent power requirement would not be needed as compared to previous OTA based electronically tunable realizations; moreover, this conversion involves thermal voltage (*V*_*T*_) that may ensue temperature sensitivity issue [18]. Also, use of the superior quality devices [28, 32] in the proposed topology is believed to exhibit low distortion generation at extended range of frequencies. A comparative study of similar designs, presented in a concise table, indicates the superiority of the proposed implementation.

As further study, we plan to utilize the linear VCQO for some cognitive radio spectrum assessment applications after translating the proposed design using suitable building blocks with appropriate high frequency specification.

## Figures and Tables

**Table 1:** 

*f* (MHz)	1	2	3

*θ*_*e*_°	1.2	2.4	3.6

**Table 2:** Comparative summary on the characteristics of some recent quadrature oscillators.

Reference	Device used	Electronic tunability	*f*_*o*_ (KHz) reported	Linear tuning law	THD (%)
[9]	VOA	No	160	No	No mention
[10]	CFA	Yes	80	Yes	2.0
[12]	CDBA	Yes	900	No	3.1
[11]	CFOA	No	159	No	3.16
[14]	CDTA	Yes	1730	No	3.0
[15]	DDCC	No	1060	No	No mention
[16]	CCCCTA	Yes	1100	No	1.15~2.94
[13]	CDBA	No	16	No	1.94
[17]	OTA	Yes	64	Yes	No mention
[18]	DVCCTA	Yes	348	No	4.66
[19]	DVCCTA	Yes	3183	No	No mention
Proposed	CFA-MMCC	Yes	1800	Yes	2.1

Frequency stability Ψ = 1 in proposed design; also reported Ψ = 1 in [10]; no mention of Ψ in other references above.

## References

[1] Chiang H. H. (1986). *Electronic Waveforming and Processing*.

[2] Lee J.-L., Liu S.-I. (2001). Integrator and differentiator with time constant multiplication using current feedback amplifier. *Electronics Letters*.

[3] Minaei S., Topcu G., Çiçekoğlu O. (2003). Active only integrator and differentiator with tunable time constants. *International Journal of Electronics*.

[4] Tanaka M., Ikeda M., Ikeda H., Inaba S., Fujita Y. Monolithic current integrator circuit as a building block of wide dynamic range ADC for calorimetry system.

[5] Nandi R., Debroy S. K. (1997). Voltage-controlled dual-input integrator with square-law enhanced time constant and its digital control. *Frequenz*.

[6] Nandi R., Goswami A., Nagaria R. K., Sanyal S. K. (2003). Voltage tunable differential integrator and differentiator using current feedback amplifiers. *IEICE Transactions on Electronics*.

[7] Tsukutani T., Tsunetsugu H., Sumi Y., Yabuki N. (2010). Electronically tunable first-order all-pass circuit employing DVCC and OTA. *International Journal of Electronics*.

[8] von Wangenheim L. (2012). Phase margin determination in a closed-loop configuration. *Circuits, Systems, and Signal Processing*.

[9] Horng J.-W. (2011). Quadrature oscillators using operational amplifiers. *Active and Passive Electronic Component*.

[10] Bhaskar D. R., Senani R., Singh A. K. (2010). Linear sinusoidal VCOs: new configurations using current-feedback-op-amps. *International Journal of Electronics*.

[11] Lahiri A., Jaikla W., Siripruchyanun M. (2013). First CFOA-based explicit-current-output quadrature sinusoidal oscillators using grounded capacitors. *International Journal of Electronics*.

[12] Nandi R., Kar M., Das S. (2009). Electronically tunable dual-input integrator employing a single CDBA and a multiplier: voltage controlled quadrature oscillator design. *Active and Passive Electronic Components*.

[13] Tangsrirat W., Prasertsom D., Piyatat T., Surakampontorn W. (2008). Single-resistance-controlled quadrature oscillator using current differencing buffered amplifiers. *International Journal of Electronics*.

[14] Jin J., Wang C. (2012). Single CDTA-based current-mode quadrature oscillator. *AEU—International Journal of Electronics and Communications*.

[15] Kumngern M. Versatile voltage-mode quadrature oscillator circuit using DDCCs.

[16] Sa-Ngiamvibool W., Jantakun A. (2014). Quadrature oscillator using CCCCTAs and grounded capacitors with amplitude controllability. *International Journal of Electronics*.

[17] Kumwachara K., Surakampontorn W. (2003). An integrable temperature-insensitive gm-RC quadrature oscillator. *International Journal of Electronics*.

[18] Lahiri A., Jaikla W., Siripruchyanun M. (2010). Voltage-mode quadrature sinusoidal oscillator with current tunable properties. *Analog Integrated Circuits and Signal Processing*.

[19] Chen H. P., Wang S. F., Hsieh M. Y. (2014). Tunable current-mode and voltage-mode quadrature oscillator using a DVCCTA. *IEICE Electronics Express*.

[20] Biolek D. CDTA—building block for current mode analog signal processing.

[21] Gift S. J. G., Maundy B. (2014). Versatile composite amplifier configuration. *International Journal of Electronics*.

[22] Bailey D. (2003). *Practical Radio Engineering & Telemetry for Industry*.

[23] Yoder S., Ismail M., Khalil W. (2011). *VCO Based Quantizers Using Frequency-to-Digital and Time-to-Digital Converters*.

[24] Liang S., Redman-White W. A linear tuning ring VCO for spectrum monitor receiver in cognitive radio applications.

[25] Bhushan M., Newcomb R. (1967). Grounding of capacitors in integrated circuits. *Electronics Letters*.

[26] (1990). *Linear Products Data Book*.

[27] (1992). *Macromodel of AD 844 AN in PSPICE Library*.

[28] Tammam A. A., Hayatleh K., Ben-Esmael M., Terzopoulos N., Sebu C. (2014). Critical review of the circuit architecture of CFOA. *International Journal of Electronics*.

[29] Gift S. J. G., Maundy B. (2005). Improving the bandwidth gain-independence and accuracy of the current feedback amplifier. *IEEE Transactions on Circuits and Systems II: Express Briefs*.

[30] Hwang Y.-S., Liu W.-H., Tu S.-H., Chen J.-J. (2009). New building block: multiplication-mode current conveyor. *IET Circuits, Devices and Systems*.

[31] Nandi R., Venkateswaran P., Kar M. (2014). MMCC based electronically tunable allpass filters using grounded synthetic inductor. *Circuits and Systems*.

[32] News Updates (2004). *Global Signal Processing Times*.

[33] Tammam A. A., Hayatleh K., Lidgey F. J. Novel high performance current-feedback op-amp.

